# Impact of single-heterozygous UGT1A1 on the clinical outcomes of irinotecan monotherapy after fluoropyrimidine and platinum-based combination therapy for gastric cancer: a multicenter retrospective study

**DOI:** 10.1007/s10147-020-01720-y

**Published:** 2020-07-14

**Authors:** Shintaro Nakano, Satoshi Yuki, Yasuyuki Kawamoto, Hiroshi Nakatsumi, Takayuki Ando, Shinya Kajiura, Ayumu Yoshikawa, Kazuaki Harada, Kazuteru Hatanaka, Aya Tanimoto, Atsushi Ishiguro, Takuya Honda, Masayoshi Dazai, Takahide Sasaki, Naoya Sakamoto, Yoshito Komatsu

**Affiliations:** 1 Department of Gastroenterology and Hepatology, Hokkaido University Hospital, Kita14, Nishi5, Kita-Ku, Sapporo, Hokkaido 060-8648 Japan; 2 Division of Cancer Center, Hokkaido University Hospital, Kita14, Nishi5, Kita-Ku, Sapporo, Hokkaido 060-8648 Japan; 3 Department of Gastroenterology and Hematology, Faculty of Medicine, University of Toyama, 2630, Sugitani, Toyama-shi, Toyama, 930-0194 Japan; 4 Department of Medical Oncology, Kushiro Rosai Hospital, 13-23 Nakazono-cho, Kushiro, Hokkaido 085-8533 Japan; 5 Department of Gastroenterology, Hakodate Municipal Hospital, 1-10-1 Minatomachi, Hakodate, Hokkaido 041-8680 Japan; 6 Department of Medical Oncology, Teine Keijinkai Hospital, 1-40, Maeda1-12, Teine-Ku, Sapporo, Hokkaido 006-8555 Japan; 7 Division of Clinical Oncology Center, Nagasaki University Hospital, 1-7-1, Sakamoto, Nagasaki 852-8501 Japan; 8 Department of Gastroenterology, Sapporo Medical Center NTT EC, Minami1, Nishi5, Chuo-Ku, Sapporo, Hokkaido 060-0061 Japan; 9 Department of Internal Medicine, Hokkaido Gastroenterology Hospital, 2-10, Honcho1-1, Higashi-Ku, Sapporo, Hokkaido 065-0041 Japan

**Keywords:** Gastric cancer, Irinotecan, UGT1A1

## Abstract

**Background:**

It is unclear whether the UGT1A1 status, single heterozygous (SH) or wild type (WT), is associated with the efficacy and toxicity of irinotecan monotherapy in advanced gastric cancer (AGC). We investigated the association between clinical outcomes (efficacy and safety) and UGT1A1 status in patients who received irinotecan monotherapy.

**Methods:**

We evaluated AGC patients who received irinotecan monotherapy between January 2011 and December 2017. Efficacy was assessed according to overall survival (OS) and progression-free survival (PFS). Toxicity was graded using the Common Toxicity Criteria for Adverse Events (version 4.0).

**Results:**

A total of 100 patients were evaluated (62 and 38 patients with UGT1A1 WT and SH, respectively). In the WT and SH groups, the irinotecan dose was reduced in 19 (30.6%) and 18 (47.2%) patients (*p* = 0.135), respectively; treatment was delayed due to adverse events (AEs) in 19 (30.6%) and 13 (34.2%) patients (*p* = 0.826), respectively; the median PFS was 3.15 and 3.25 months (HR, 0.734; 95% CI 0.465–1.158; *p* = 0.184), respectively; and the median OS was 10.4 and 7.26 months (HR, 1.137; 95% CI 0.752–1.721; *p* = 0.543), respectively. Severe hematological AEs (Grade ≥ 3) were significantly more frequent in the SH group than in the WT group (63% vs. 36%; *p* = 0.008), while severe non-hematological AEs was not significantly different (16.0% vs. 6.5%; *p* = 0.173).

**Conclusion:**

There was no significant difference in the efficacy of irinotecan monotherapy between UGT1A1 WT and UGT1A1 SH, but UGT1A1 SH was associated with a high frequency of severe hematological toxicity.

## Introduction

Gastric cancer is the third leading cause of cancer-related mortality worldwide, accounting for 8.8% of all cancer deaths [1]. Advanced gastric cancer (AGC) is primarily treated with systemic chemotherapy, with fluoropyrimidine and platinum-based combination therapy recommended by several guidelines [2, 3] as first-line chemotherapy. Irinotecan is also one of the important treatment options for gastric cancer and is mainly used as a second- or later-line chemotherapy. However, the survival benefit of this treatment is yet to be established. While it was reported to improve overall survival (OS) compared to best supportive care [4, 5], it was not superior to paclitaxel in the randomized phase III trial, WJOG4007 [6], but rather showed similar efficacy. The Pan-Asian-adapted ESMO Clinical Practice Guidelines recommend second-line chemotherapy with either irinotecan, taxanes, ramucirumab monotherapy, or combination ramucirumab and paclitaxel therapy for metastatic gastric cancer [7]. The latest Japanese gastric cancer treatment guideline (5^th^ edition) recommends irinotecan monotherapy as a third-line chemotherapy [3].

Irinotecan is a pro-drug that is converted to SN-38, which is an activated form and acts as a topoisomerase I inhibitor in the liver [8]. SN-38 is inactivated by the liver enzyme UGT1A1 [9] to SN-38 glucuronide (SN-38G). Several studies showed that UGT1A1 polymorphisms (homozygous or double heterozygous UGT1A1*6/*28) is associated with delayed metabolism of SN-38, and this leads to enhanced irinotecan-induced toxicity in various tumors including gastric cancer [10–13]. Ethnic differences in the frequency of the UGT1A1*6 variant have been reported, with higher frequency in Asians than in Caucasians (13–17% vs. < 0.1%) [14–17]. Homozygous UGT1A1*6 is also associated with severe neutropenia [16]. However, it remains unclear whether single heterozygous UGT1A1 (SH) affects the efficacy and safety of irinotecan monotherapy compared to wild type UGT1A1 (WT). Thus, this study aimed to evaluate the efficacy and safety of irinotecan as second- or later-line chemotherapy according to UGT1A1 status in AGC patients refractory or intolerant to fluoropyrimidines and platinum.

## Materials and methods

### Patients and study design

We retrospectively reviewed the clinical data of patients with AGC who received irinotecan monotherapy between January 2011 and December 2017 in any of the 8 participating institutions (Hokkaido University Hospital, University of Toyama Hospital, Kushiro Rosai Hospital, Hakodate Municipal Hospital, Teine Keijinkai Hospital, Nagasaki University Hospital, Sapporo Medical Center NTT EC, and Hokkaido Gastroenterology Hospital) in Japan. The eligibility criteria were age ≥ 20 years, histologically or cytologically confirmed gastric cancer, received irinotecan monotherapy as second- or later-line treatment, and refractory or intolerant to fluoropyrimidine and platinum. Patients were excluded if they were treated with cytotoxic triplet regimen and if the irinotecan dose was initially reduced. The study design and protocol were approved by the institutional review board of Hokkaido University Hospital and all other participating institutions. The need for informed consent was waived owing to the retrospective nature of the study. This research was announced on a website (https://www.huhp.hokudai.ac.jp/).

### Irinotecan monotherapy

Irinotecan, 150 mg/m^2^ of body surface area, was administrated intravenously within 120 min every 2 weeks. The treatment was continued until disease progression, occurrence of unacceptable adverse effects, or patient’s refusal to continue.

### Outcome assessment

Efficacy was assessed using OS (defined as the time from the start of first irinotecan administration to death) and progression-free survival (PFS; defined as no progression at the time of survival investigation). Patients whose treatment regimens were changed without evidence of progression were censored. Tumor response was evaluated according to the Response Evaluation Criteria in Solid Tumors (ver. 1.1). Toxicity was graded using the Common Toxicity Criteria for Adverse Events (ver. 4.0).

### Statistical analysis

Qualitative and quantitative variables were compared using the chi-square test or Fisher’s test and using a nonparametric (Wilcoxon) test, respectively. Data were presented with 95% confidence intervals calculated using standard methods based on a binomial distribution. Survival analyses were performed with Kaplan–Meier method. A log-rank test and a Cox proportional hazard model were used to compare patients according to UGT1A1 status (WT vs. SH type). Both efficacy and safety analyses included all patients who received at least one dose of irinotecan. All analyses were performed using SPSS ver. 25 (IBM, Armonk, NY, USA). All tests were two-sided, and a *p* value of < 0.05 was considered statistically significant.

## Results

### Patient characteristics

**Fig. 1:**
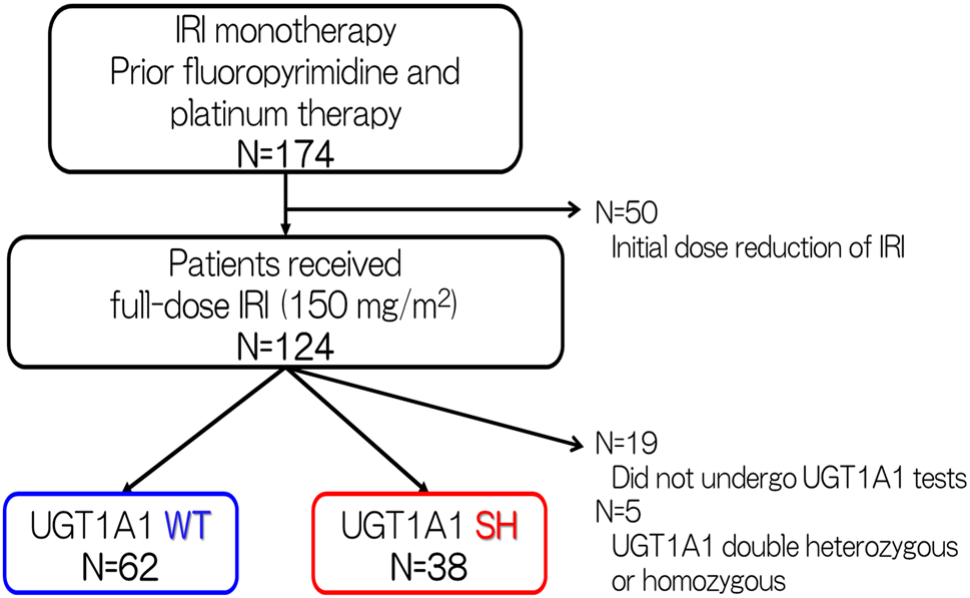
Flow diagram for patient inclusion. *IRI* irinotecan; *WT* wild type, *SH* single heterozygous

Of the 174 patients initially assessed, 74 patients were excluded because the irinotecan dose was initially reduced (*n* = 50), they did not undergo UGT1A1 tests (*n* = 19), or they had a UGT1A1 double-heterozygous or homozygous status (*n* = 5; Fig. 1). Finally, 100 patients were included in the analysis. Among them, 62 and 38 patients were UGT1A1 WT and UGT1A1 SH, respectively.

In the UGT1A1 WT and SH groups, 60 (97%) and 34 (90%) patients had an Eastern Cooperative Oncology Group performance status of 0 or 1, 14 (23%) and 9 (24%) patients had 3 or more metastatic sites, 17 (27%) and 8 (21%) patients had HER2-positive status, 46 (74%) and 30 (79%) patients received taxanes as prior therapy, and 27 (44%) and 18 (47%) patients received irinotecan as second-line chemotherapy, respectively. The patients’ clinicopathological characteristics are listed in Table 1.

**Table 1:** Patient characteristics according to UGT1A1 groups

Variables	WT group *n* = 62	SH group *n* = 38	*p* value
Sex, *n* (%)			1.000^*^
Male	49 (79.0)	30 (78.9)	
Female	13 (21.0)	8 (21.1)	
Age (years), median (range)	67 (22–81)	64 (31–83)	0.323^**^
ECOG-PS, *n* (%)			0.423^*^
0	20 (32.3)	11 (29.0)	
1	40 (64.5)	23 (60.5)	
≥ 2	2 (3.2)	4 (10.5)	
Pathology, *n* (%)			0.067^*^
Intestinal	38 (61.3)	16 (42.1)	
Diffuse	24 (38.7)	22 (57.9)	
Synchronous metastases, *n* (%)			1.000^*^
Yes	53 (85.5)	32 (84.2)	
No	9 (14.5)	6 (15.8)	
Number of metastatic organs, *n* (%)			0.580^*^
1	22 (35.5)	10 (26.3)	
2	26 (41.9)	19 (50.0)	
≥ 3	14 (22.6)	9 (23.7)	
HER2 status, *n* (%)			0.635^*^
Positive	17 (27.4)	8 (21.1)	
Negative	45 (72.6)	30 (78.9)	
Prior therapies, *n* (%)			0.708^*^
1	27 (43.5)	18 (47.4)	
2	30 (48.4)	19 (50.0)	
≥ 3	5 (8.1)	1 (2.6)	

*WT* wild type, *SH* single heterozygous, *ECOG-PS* Eastern Cooperative Oncology Group-performance status, *HER2* human epidermal growth factor receptor 2

^*^Fisher’s exact test

^**^Mann–Whitney test

### Treatment exposure

By the cut-off date of September 30, 2018, all 100 patients had discontinued irinotecan treatment with a median follow-up time of 7.9 months. In the WT and SH groups, dose reduction of irinotecan was required in 19 (30.6%) and 18 (47.2%) patients (*p* = 0.135), and treatment was delayed due to adverse events (AEs) in 19 (30.6%) and 13 (34.2%) patients (*p* = 0.826), respectively. The median treatment cycle was 6 and 4 (*p* = 0.278), and the median relative dose intensity was 82% and 80% (*p* = 0.864), respectively. In total, 58 (93%) and 35 (92%) patients discontinued irinotecan treatment because of disease progression, and 40 (65%) patients in the UGT1A1 WT group and 27 (71%) patients in the UGT1A1 SH group received subsequent chemotherapy after treatment failure with irinotecan.

### Efficacy

**Fig. 2:**
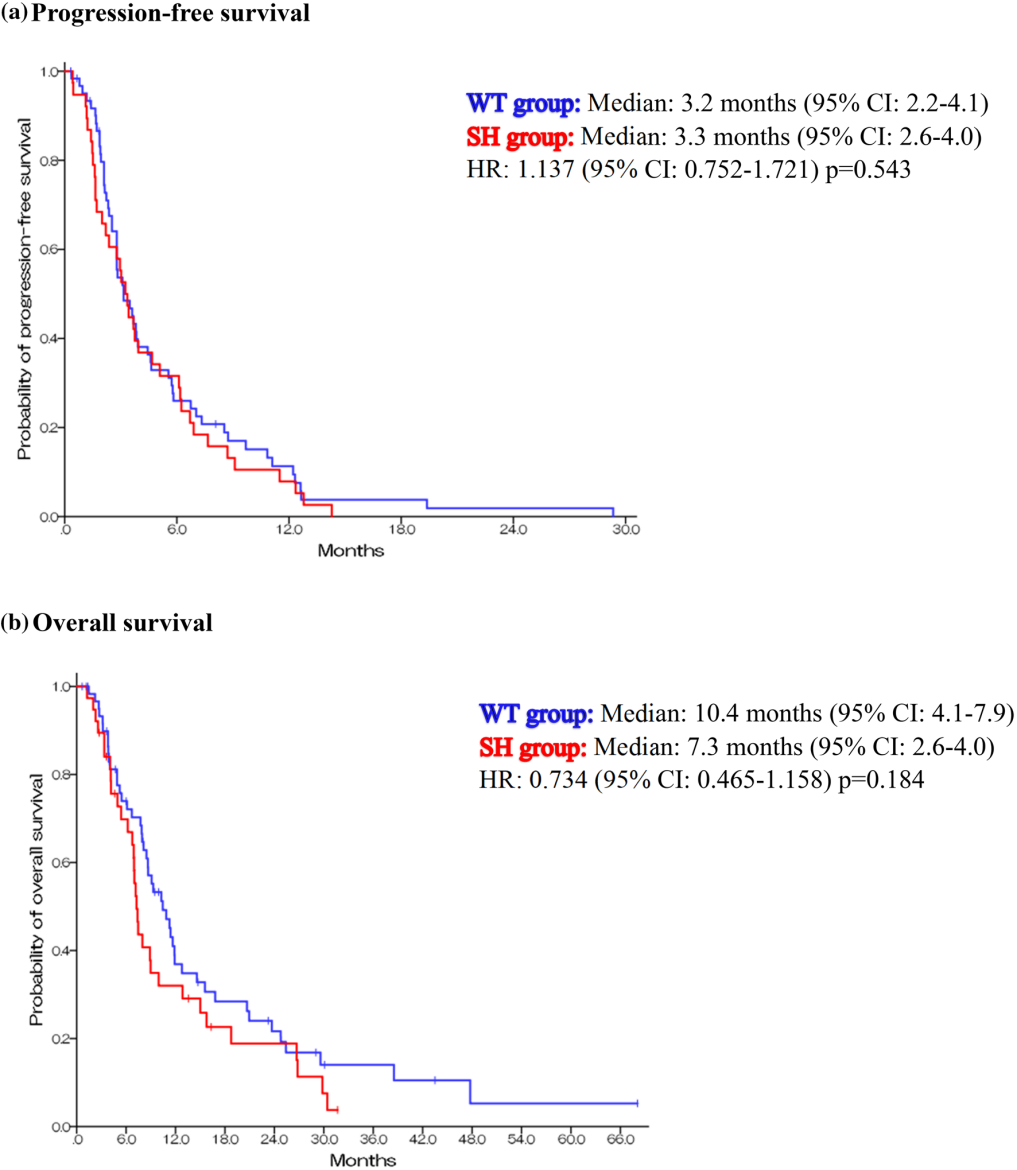
Kaplan–Meier survival analysis. *WT* wild type, *SH* single heterozygous

Treatment response to irinotecan monotherapy is summarized in Table 2. Of the 100 patients, 88 patients had measurable lesions. The objective response rate was 13.0% in the UGT1A1 WT group and 2.9% in the UGT1A1 SH group (*p* = 0.145). The disease control rate was 61.1% and 41.1% (*p* = 0.131), respectively. The median PFS was 3.2 months (95% CI 2.21–4.09) in the UGT1A1 WT group and 3.3 months (95% CI 2.56–3.95) in the UGT1A1 SH group (HR, 1.137; 95% CI 0.752–1.721; *p* = 0.543) (Fig. 2a). The median OS was 10.4 months (95% CI 7.91–13.0) in the UGT1A1 WT group and 7.26 months (95% CI 6.61–7.92) in the SH group (HR 0.734; 95% CI 0.465–1.158; *p* = 0.184) (Fig. 2b).

**Table 2:** Treatment response to irinotecan according to the UGT1A1 group

	N	CR	PR	SD	PD	NE	RR (%)	DCR (%)
WT group	54	0	7	26	18	3	13.0	61.1
SH group	34	0	1	14	19	0	2.9	44.1

*CR* complete response, *DCR* disease control rate, *N* number, *NE* not evaluated, *PD* progressive disease, *PR* partial response, *RR* response rate, *SD* stable disease, *SH* single heterozygous, *WT* wild type

### Safety analysis

The AEs related to irinotecan are shown in Table 3. No patients died of treatment-related causes in either group. Severe (grade 3 or 4) hematological AEs were significantly more frequent in the UGT1A1 SH group than in the UGT1A1 WT group (63% vs. 36%; *p* = 0.008), whereas there was no significant difference in the frequency of non-hematological AEs between the two groups (16% vs. 6.5%; *p* = 0.173). Among hematological AEs, the incidence of neutropenia was higher in the UGT1A1 SH group than in the UGT1A1 WT group, but the difference was not significant (32% vs. 15%; *p* = 0.109). The common (5% or higher) severe AEs were decreased white blood cell count (11% and 16%), neutropenia (15% and 32%), anemia (21% and 24%), and anorexia (1.6% and 11%) in the UGT1A1 WT and UGT1A1 SH groups.

**Table 3:** Safety profile of irinotecan according to the UGT1A1 group

		WT group (*n* = 62)		SH group (*n* = 38)		*p* value^*^	
Adverse events		All grades *n* (%)	Grade ≥ 3 *n* (%)	All grades *n* (%)	Grade ≥ 3 *n* (%)	All grades	Grade ≥ 3
*Hematological events*		51 (82)	22 (36)	35 (92)	24 (63)	0.238	0.008
Leukopenia		38 (61)	7 (11)	25 (66)	6 (16)	0.391	0.750
Neutropenia		32 (52)	9 (15)	24 (63)	12 (32)	0.283	0.109
Anemia		31 (50)	13 (21)	23 (61)	9 (24)	0.331	0.826
Thrombocytopenia		12 (19)	1 (1.6)	5 (13)	1 (2.6)	0.654	0.929
*Non-hematological events*		55 (89)	4 (6.5)	33 (87)	6 (16)	0.762	0.173
Febrile neutropenia		1 (1.6)	1 (1.6)	0 (0)	0 (0)	1.000	1.000
Nausea		28 (45)	0 (0)	16 (42)	1 (2.6)	0.837	0.380
Vomiting		11 (18)	0 (0)	4 (11)	1 (2.6)	0.397	0.380
Anorexia		35 (57)	1 (1.6)	16 (42)	4 (11)	0.217	0.067
Fatigue		33 (53)	0 (0)	21 (55)	3 (7.9)	1.000	0.052
Diarrhea		28 (45)	2 (3.2)	19 (50)	1 (2.6)	0.683	1.000
Alopecia		16 (26)	-	16 (42)	-	0.122	-

*WT* wild type, *SH* single heterozygous

^*^Fisher’s exact test

## Discussion

This multicenter retrospective study showed that among AGC patients treated with irinotecan monotherapy as second- or later-line treatment the incidence of hematological AEs was higher in UGT1A1 SH patients than UGT1A1 WT patients. Several studies have indicated that UGT1A1 polymorphism is associated with toxicity in irinotecan monotherapy [11, 12, 13, 19] and combination therapy [10, 12, 19–21]. Racial differences in UGT1A1 polymorphism have also been reported. Marsh et al. reported a lower frequency of the UGT1A1*28 variant in Asian patients than in Caucasian patients. Meanwhile, the UGT1A1*6 variant is higher in Asian patients than in Caucasian patients [22]. Homozygous or double-heterozygous UGT1A1*6 or *28 is associated with higher incidence of severe neutropenia but not diarrhea [10, 11]. Yamaguchi et al. [13] reported that gastric cancer patients who have double heterozygous or homozygous UGT1A1 tend to have severe neutropenia. However, the association of single-heterozygous and wild type UGT1A1 with hematological toxicity is unclear. Nishimura et al. [23] reported a higher incidence rate of hematological toxicity in irinotecan monotherapy as third-line treatment for AGC refractory to fluoropyrimidines, platinum, and taxanes. Yamaguchi et al. [13] reported a similar rate of toxicity in third-line irinotecan monotherapy between UGT1A1 WT and SH patients. The discrepancy may be explained by the different rates of initial dose reduction of irinotecan. In our study, the irinotecan dose was initially reduced in 28.7% (50/174) of patients. Therefore, we only included the patients who received irinotecan monotherapy at 150 mg/m^2^, which is widely regarded as a full dose in Japan, during the first cycle.

Our study clearly shows that UGT1A1 SH was associated with a higher incidence rate of severe hematological toxicity, mainly because of the increasing rate of neutropenia. This trend was also shown regardless of treatment line and use of taxanes. Non-hematological toxicity such as diarrhea did not significantly differ according to UGT1A1 status, consistent with the findings of previous studies [13, 23, 24]. To date, few studies and guidelines have mentioned the impact of UGT1A1 SH on the risk of AEs in irinotecan monotherapy. However, clinicians should be aware that not only double heterozygous or homozygous UGT1A1, but also SH is a significant risk factor for severe hematological AEs.

With respect to efficacy, we found that the UGT1A1 status did not influence the PFS in irinotecan monotherapy. The PFS rate in the current study was similar to those in previous reports. The PFS rate in irinotecan monotherapy as second- or later-line treatment ranged from 2.2 to 4.1 months [6, 13, 23–26]. Yamaguchi et al. [12] showed that UGT1A1 double heterozygous and homozygous were associated with poor outcomes. However, it remains unclear whether UGT1A1 SH affects the efficacy of irinotecan. Our study showed that the OS of patients with UGT1A1 SH seemed worse than that of those with UGT1A1 WT, although this was not statistically significant. Even though the PFS was similar, OS was different; this discrepancy of OS and PFS may be attributable to differences in post-irinotecan treatment. One speculation is that a higher incidence of severe hematological adverse events might lead to a lower dose intensity of post-irinotecan treatment. However, we do not have sufficient data to evaluate this speculation.

Recently, nivolumab [27] and trifluridine/tipiracil (TAS-102) [28] were approved for the treatment of AGC, and an emerging clinical question is which drug must be chosen for patients with AGC who need salvage line treatment. Our results revealed that in patients with UGT1A1 WT, irinotecan monotherapy is comparable with nivolumab treatment, which is an expensive drug. Kato et al. reported that the response rate for nivolumab treatment was higher than that for irinotecan treatment in slow-growing tumors, whereas the response rates of these two drugs were comparable in rapid-growing tumors [29]. The effects of TAS-102 are modest, as the response rate was 4%; hence, TAS-102 may be less effective for treating rapidly progressing tumors. Thus, treatment with irinotecan may be a better alternative for patients with UGT1A1 WT or when tumor progression is rapid.

Our study has several limitations. First, the inherent biases in a retrospective study could not be eliminated. However, we tried to decrease the bias by collecting many patients from several institutions. To our knowledge, our study is the largest retrospective study to analyze the impact of UGT1A1 status on the efficacy and safety of irinotecan monotherapy in AGC. Second, many novel drugs (such as oxaliplatin, nab-paclitaxel, ramucirumab, nivolumab, and TAS-102) have been approved for gastric cancer in Japan during the study period, and this has influenced the guidelines and clinical practice.

In conclusion, there was no significant difference in the efficacy of irinotecan monotherapy according to the UGT1A1 status. However, UGT1A1 SH patients showed a higher incidence of severe hematological AEs in bi-weekly irinotecan monotherapy. Clinicians should be aware of the risk when treating these patients with irinotecan. Further well-designed, large-scale prospective studies are needed to clarify the association between UGT1A1 SH and risk of hematological AEs.
